# (In)consistency matters: An account of understanding the perception of inconsistent expressions on social media

**DOI:** 10.3389/fpsyg.2022.885498

**Published:** 2022-08-04

**Authors:** Pengxiang Li, Hichang Cho, Yuren Qin

**Affiliations:** ^1^School of Journalism and Communication, Minzu University of China, Beijing, China; ^2^Department of Communications and New Media, Faculty of Arts and Social Sciences, National University of Singapore, Singapore, Singapore; ^3^College of Media and International Culture, Zhejiang University, Hangzhou, China

**Keywords:** perceived consistency, authenticity, warranting, interpersonal perception, computer-mediated communication

## Abstract

In their daily use of social media, most people cannot maintain consistency in every message they present, leading observers to experience a feeling of inconsistency. Building on computer-mediated interpersonal theories [i.e., attribution theory, warranting theory, and authenticity model of computer-mediated communication (CMC)], this study aims to explore how people interpret and reconcile perceived inconsistent expressions on social media. Through thematic analysis of data obtained from six focus groups, two main themes were extracted: the origin of perceived inconsistency on social media and the strategies for reconciling perceived inconsistency. Specifically, three forms of perceived inconsistent information were identified: those within the same account; those between public and private accounts; and those between online and offline settings. Additionally, three types of reconciliation strategies were distilled from participants’ narratives: relying on authentic representation; engaging in perspective-taking to compensate for situational factors; and inferring inner motives behind acting inconsistently. With these two themes, this study proposes a two-stage model of processing inconsistency (i.e., reasoning from inconsistency to consistency) in CMC. This model suggests that several factors–including perceived authenticity, social categorical cues, and relationship or familiarity between observers and a presenter–are involved in perceiving inconsistent information and determine the outcomes of interpersonal evaluations. These findings enhance our understanding of online interpersonal perceptions.

## Introduction

The way of forming interpersonal impressions has been greatly altered in computer-mediated communication (CMC), particularly in the era of social media. People chronicle their daily lives on social media and leave numerous media traces–whether a text, photograph, video clip, or even an emoji (Humphreys, 2018), which, in turn, serve as social cues for observers to learn about that person’s characteristics or personality (Hall et al., 2013). In today’s social media context, it is common to see inconsistencies between such cues because people cannot remember every piece of trace they left, or they may intend to do so (Tang et al., 2020). A typical example of self-contradiction is former President Trump on Twitter, who constantly conveyed inconsistent messages on various issues such as foreign policies or the coronavirus crisis (Shane, 2018; The Washington Post, 2020). Despite the prevalence of inconsistent presentation online, the impact of perceived inconsistency on interpersonal perception remains unclear. Theoretically, inconsistent information violates the normative expectation of coherence and consistency in impression formation and could result in negative evaluations of the target person (Asch and Zukier, 1984; Hampson, 1998). Supporting this theoretical presumption, several studies found that one’s inconsistent expressions negatively affect that person’s trustworthiness, attractiveness, or authenticity (e.g., DeAndrea and Walther, 2011; Hong et al., 2012; Tang et al., 2020), but other studies suggest that a certain level of inconsistency in one’s online performances may not necessarily harm his/her impression (e.g., Lee et al., 2020; Qin et al., 2021).

The mixed findings of the influence of perceived (in)consistency have led to a demand for further exploration. With this said, a comprehensive exploration of the underlying process of perceiving inconsistent information is essential to learn which factors are involved in this process and shape the outcomes of interpersonal evaluations. It is also of great social significance to study the perception of (in)consistent expressions in CMC. The unique characteristics of CMC–lack of socio-contextual cue and ubiquitous selectivity and editability of presentation–have reconfigured the way people perceive one another (Toma and Hancock, 2010). Observers thus seek information that is deemed normative to form a stable impression of a target person and take non-normative performances or unexpected behaviors into serious consideration to avoid potentially negative outcomes (e.g., deception) when developing interpersonal rapports. As such, it is important to study how observers perceive non-normative information like inconsistent expressions to expand our knowledge of online impression formation.

Drawing on interpersonal perception theories, the present study aims to explore how people perceive and reconcile inconsistent information on social media. To this end, we conduct a series of in-depth focus groups with social media users. By analyzing the narrative data obtained from these focus groups, we propose a novel, refined theoretical model systematically presenting the process of perceiving inconsistent information online. The results advance the understanding of the nature of perceived (in)consistency and contribute to the ongoing conversation about computer-mediated interpersonal perception.

## Literature review

### Perceiving inconsistency in offline contexts

Before diving into the perception of inconsistent expressions on social media, it is enlightening to understand how people deem a set of behaviors as inconsistent in offline contexts and how they explain such inconsistencies. When perceiving others, people naturally expect internal consistency and unity from a target person’s own traits and behaviors (Greenwald, 1982; Asch and Zukier, 1984), and disagreement between two subsets of social cues from a target person can elicit difficulties in forming an interpersonal impression (Slovic, 1966). In this regard, perceived inconsistency in offline contexts describes the situation when a person’s traits or behaviors conflict with each other, violating the expectation of coherence and causing cognitive disequilibrium for observers (Hampson, 1998). To resolve this cognitive disequilibrium, observers need to reconcile and resolve perceived inconsistent cues and ultimately achieve a stable impression of the target person (Johnson-Laird et al., 2004). Extensive literature has revealed different measures that people adopt to deal with inconsistent expressions offline.

One line of research focuses on how people use valid cues to reconcile inconsistency. When two sets of cues are inconsistent, observers either utilize relatively more valid cues and exclude the rest to draw a conclusion (Slovic, 1966), or assign greater weight to more valid cues (Himmelfarb, 1970). Recent studies have examined further which types of cues are valid or invalid for social judgments. Newly added cues usually are perceived as less valid when they are inconsistent with observers’ existing knowledge about or initial impression of the target (Chung and Fink, 2016). The valence (i.e., positivity vs. negativity) of personal traits is viewed as more valid than those traits’ nature (i.e., warmth vs. competence; Brannon et al., 2017). In addition, morality-related traits are more informative than sociability- or competence-related traits (Brambilla et al., 2019). This line of research thereby indicates that when faced with inconsistency, people compare conflicting cues’ validity from different perspectives (e.g., novelty, valance, or morality) and count those that they deem more valid to address the perceived inconsistency.

The other line of research is grounded in attribution theory, which examines how people explain the causes of unexpected behavior and events in daily life (Weiner, 1985). Considering that inconsistent expressions are unexpected and non-normative, people attempt to make causal explanations for such information, and they search for either dispositional causes (internal causality) or situational causes (external causality; Jones and Nisbett, 1987; Johnson-Laird et al., 2004). Both types of causality are useful in addressing the perceived inconsistent information, but the adoption of the two strategies is contingent on the relationship between the actor and the observer. The actor-observer asymmetry of attributions–the fact that people favor themselves or close others when making attributions–influences how people process perceived inconsistency (Jones and Nisbett, 1987). For instance, observers would make dispositional attributions (e.g., forming a negative impression) when explaining the incongruence between an unfamiliar target’s verbal and non-verbal expressions (Weisbuch et al., 2010). However, compared with unfamiliar others, people do not consider their conflicting traits or behaviors as inconsistent because they believe that their own personalities are more multifaceted and complex (Sande et al., 1988; Prentice, 1990).

Taken together, existing literature indicates that different types of inconsistency lead to distinct attribution orientations (e.g., dispositional vs. situational), in which the target’s standpoint (e.g., oneself/close others vs. acquaintances) also plays a role. If the perceived inconsistency can be explained through situational factors, the target’s impression is not affected; otherwise, the impression of the target, especially for an acquaintance, is affected negatively (Hampson, 1998).

### Perceiving inconsistent information on social media

Social media have offered a perfect venue for interpersonal perceptions and impression formations. People, as observers, aggregate different media traces left by a presenter (e.g., text posts and photographs) over time and across settings to infer the presenter’s characteristics and his/her identity (Humphreys, 2018). Unlike in offline contexts, social media users not only rely on a presenter’s own statements to draw inferences but also utilize information generated by other sources (e.g., friends’ comments or system-generated information) to do so. A large body of research has examined how observers rely on valid cues on social media (e.g., status updates, profile pictures, number of friends, or friends’ appearances) to develop relatively accurate judgment about a target’s personality traits, likability, popularity, or attractiveness (e.g., Walther et al., 2008; Utz, 2010; Hall et al., 2013). These studies mainly focus on different cues working complementarily and consistently to help social media users form impressions, while the literature on perceiving inconsistent cues online is limited.

As mentioned above, observers seek information created by various sources–including the presenter, his/her friends, and the system–to form interpersonal impressions. Therefore, the forms of inconsistent expressions are diverse on social media, including inconsistency occurring within a presenter’s self-claimed statements or inconsistency generated by different sources (e.g., self-claimed statements vs. other-generated and/or system-generated statements). Warranting theory has been widely adopted to investigate the inconsistent expressions caused by different sources (e.g., the information generated by oneself, by friends or acquaintances, and by the system). According to warranting theory, people view cues with higher warranting value (i.e., the degree to which a cue or piece of information is immune to manipulation by the target) to be more valuable and choose to rely on these cues when making interpersonal evaluations (Walther and Parks, 2002). Based on this theoretical framework, previous studies have concluded that social media users attach higher warranting value to system-generated information (e.g., number of friends) and other-generated statements (e.g., friends’ comments), which is less likely to be manipulated compared with self-claimed statements (Walther et al., 2009; Utz, 2010; DeAndrea et al., 2015). In other words, when two sets of cues on social media conflict, observers assign different levels of warranting value and validity to different pieces of information, and they are inclined to trust those generated by third parties than those by the presenter.

Warranting theory may not be an ideal framework for understanding inconsistency occurring within the same person’s expressions (i.e., self-generated inconsistency) because it does not explicitly explain how observers assess the warranting value of different cues generated by the same source. Lee (2020) proposed the “authenticity model of CMC,” in which, she pointed out the significant roles of perceived consistency and authenticity in computer-mediated interpersonal perceptions. Indeed, a target person’s authenticity is dependent on the level of (in)consistency between the target’s performances and observers’ base-rate knowledge. To some extent, the authenticity model can help us understand the influence of self-generated inconsistency because it implicates that self-generated inconsistency could lead to negative interpersonal evaluations (i.e., harm the authenticity of a presenter). In line with this proposition, a recent experimental study confirmed that online presentation inconsistencies induced negative evaluations of a target person’s authenticity and likeability (Tang et al., 2020). Similarly, prior research found that participants viewed a target whose online presentations deviated from his/her offline performances (i.e., observers’ base-rate knowledge) as less trustworthy but more hypocritical (DeAndrea and Walther, 2011). In contrast, other research suggests that inconsistent performances on social media do not cause a negative impact. For instance, compared with those who constantly present positive information, people presenting both positive and negative information (inconsistent performances in a sense) on social media are perceived as reliable and trustworthy (Qin et al., 2021). This is because observers suspect that people who always present similar information are trying to manipulate or fabricate their profiles.

Existing literature reaches a preliminary conclusion regarding the influence of perceived inconsistency, which is, the inconsistent expressions on social media do not necessarily result in negative outcomes. Such a conclusion raises further theoretical queries. Which factors are involved in the process of perceiving inconsistency and determine the outcomes of interpersonal evaluations? Under what circumstances would two sets of conflicting cues on social media violate the expectation of consistency and cause a feeling of inconsistency? According to the authenticity model, observers rely on base-rate knowledge to judge whether one’s performances are consistent or not (Lee, 2020), but it is unclear how observers set base-rate knowledge about a presenter in online contexts or how they assess the credence or warranting value when two pieces of conflicting information–both generated by the same source–are equally manipulative. To address these concerns, the first research question is proposed:

RQ1: On social media, how do observers perceive inconsistent information, especially self-generated inconsistency?

It also remains unclear whether and how attribution theory can be applied in explaining perceived inconsistency on social media (Weiner, 1985; Jones and Nisbett, 1987). Given the dearth of socio-contextual cues in CMC, especially during non-spontaneous interactions (e.g., viewing Facebook timelines or Twitter and Instagram posts), how do observers attribute inconsistent expressions to situational causes like they do in offline settings? Whether or not the actor-observer asymmetry takes effects in reconciling perceived inconsistency on social media? The present study addresses these concerns by answering the second proposed research question:

RQ2: On social media, how do observers reconcile perceived inconsistent information?

## Materials and method

We conducted focus group interviews to obtain rich narratives about social media users’ thoughts and experiences in perceiving inconsistent expressions. By eliciting meaningful themes from focus group discussions, researchers can attain deep knowledge about their research topics (Strauss and Corbin, 1998; Onwuegbuzie et al., 2009).

### Data collection

Participants were recruited through a combination of purposive sampling and snowball sampling. We aimed to interact with our participants in person in order to facilitate the group discussion and attain rich narrative and interactive data, so the participants were recruited in our resided city, Singapore. The recruitment announcements were firstly posted on a local online forum in Singapore. After getting a few participants as starters, we asked them to recommend people who had experienced inconsistent expressions to enter our research project. Participants were eligible if they were over age 18 and visited any social media platforms (e.g., Facebook, Instagram, Twitter, etc.) at least once per day.

Altogether, 48 interviewees participated in the study (20 males, 28 females). To keep the diversity of participants, half of our participants were college students (including undergraduates and master students), while the other half were young professionals. Facebook and Instagram were the most popular platforms among participants, with 44 indicating daily use of the former and 42 daily use of the latter. Twitter and Snapchat also were used among participants, with 16 indicating daily use of one platform and 16 on the other. Participants’ ages ranged from 19 to 28 years old. Young people are tech-savvy and use social media more frequently compared to other age groups (Marketing Charts, 2021; Oberlo, 2021). Most of their daily interactions (e.g., building relationships, meeting new friends, and/or keeping social connections) occur on social media, so inconsistent expressions are commonly visible on young people’s social media profiles. That said, the relatively young age group can provide abundant narratives and specific personal stories regarding inconsistent expressions on social media which can help us explore the phenomenon more deeply and comprehensively.

Six focus groups (the group size ranging from six to nine) were conducted in Singapore from April 2018 to January 2019. The focus group sessions lasted 60–120 min each, depending on group size. Each session was audio-recorded and transcribed for data analysis. Two facilitators administrated the focus groups, with one moderating the discussion and the other taking notes. All the group discussions were accomplished in a lab at a public university in Singapore. Upon arrival, discussants were asked to read and sign a consent form to indicate their voluntary participation. They were also assigned a pseudonym respectively to anonymize the identifiable information. After which, the moderator introduced the purpose of the study and asked discussants to introduce their daily use of social media, including the frequently used platforms, the purposes, and the routines of using each platform, and the social interactions that occurred on different platforms. Sequentially, the moderator began to guide to discussants to enter the focus of this study and facilitated them to share their personal experiences. In particular, semi-structured questions were used to guide the group discussion. These questions explored how participants defined a particular set of expressions as inconsistent, how they noticed and reacted to such expressions, under what conditions inconsistent expressions affected their judgments, and how they perceived different social actors’ inconsistent expressions (for details, see Appendix). The discussions were not limited to these questions, as the facilitators probed more deeply into certain topics when inspired by the conversations.

### Data analysis

Constant comparison analysis, which uses an emergent-systematic approach to extract and identify themes, was employed to help researchers effectively identify the theoretical saturation of the data (Onwuegbuzie et al., 2009). Both facilitators reviewed and analyzed the transcribed recordings after each focus group session. Emergent themes from earlier groups were used for exploratory purposes, and data from later groups were used to verify and refine themes systematically. Using this approach, we ceased data collection after six focus groups, as adequate data had been collected to conduct a detailed analysis. The two facilitators worked together to code data and held iterated discussions to reach agreements on the extracted codes and themes that appropriately expressed participants’ meanings. The data analysis process followed three coding stages: open coding (in which transcripts were chunked and isolated into small units of individual phrases or verbatim quotes); axial coding (in which the units identified in the first stage were classified into different groups according to their conceptual similarities); and selective coding (in which the groups created in the second stage were organized based on meaningful dimensions) (Strauss and Corbin, 1998). Two main themes that reflect the group conversations’ content were then proposed.

## Results

Two main themes emerged from our data analysis: the origin of perceived inconsistency and the reconciliation strategies for perceived inconsistency (for the summary of research findings, see Table 1). The first theme–the origin of perceived inconsistency–unveiled that an inconsistent impression is formed when a presenter’s expression is perceived as deviating from his/her authentic representation (i.e., when a presenter is perceived as real and true; Lee, 2020). The results showed that observers adopt different criteria to determine a presenter’s authentic representation across contexts, which further influences whether they obtain feelings of inconsistency from a presenter’s expressions. Indeed, perceived inconsistency can be identified under three conditions: within the same social media account; between public and private social media accounts; and between online and offline contexts.

**TABLE 1:** Summary of research findings.

Themes		Definitions	Sample statements
**Origin of perceived inconsistency**		Expressions deviating from or threatening one’s authentic representation	
*Sub-themes*	Inconsistency within the same account	Originating from discrepancies between one’s behavioral performances and beliefs/inner feelings	She posted and reposted lots of articles supporting pro-environmental behaviors on FB, like taking public transportation or never using animal products. The funny thing is, a few days ago, I saw she posted a pic [in which she] held a leather purse and said, “new bag, yay!”
	Inconsistency between public and private accounts	Originating from discrepancies between the desirable image/identity one wants to build on a public account and the inner thoughts/feelings one expresses on a private account	For their main [public] account, they show they are having fun, but, in their private account, they would say, “I’m depressed, this thing is happening, blah blah.” I think it is incongruent in a sense.
	Inconsistency between online and offline	Originating from discrepancies between one’s persona in real life offline and his/her online performances	The guy is very quiet and passive offline. But online, like on Twitter, he’s very chatty, always sharing opinions about political or social issues like cyberbullying.
**Reconciliation strategies for inconsistency**		Reconciling perceived inconsistency to resolve cognitive disequilibrium and attain a stable impression	
*Sub-themes*	Relying on authentic representation	Attaching greater credence to cues reflecting one’s authentic aspects but devaluing other conflicting information	Posts in private accounts feel like it’s “personal human being” there, but on public [accounts], everyone looks the same. Sometimes, people even say more true and deep things on private account [than offline]. So when the two [public vs. private] conflict, I’d rather trust the private one.
	Perspective-taking	Attributing inconsistency to external/situational factors by perspective-taking	I think they [his friends] have reasons to do certain things at that given condition, [so] it [performing inconsistently] is really not their fault.
	Inferring inner motives	Seeking internal/dispositional causes for why one acting incongruently	Even I don’t know [that person], it’s easy to tell how much a [college] student earns or spends a month. Then how can a student support a life of going parties every day, or good restaurant? They [are] doing so just cause they want to attract more followers [on Instagram].

The second theme uncovered three strategies for reconciling perceived inconsistency: relying on authentic representation; perspective-taking to compensate for situational factors, and inferring inner motives behind incongruence. The first strategy suggests that cues revealing a presenter’s authentic representation are perceived to be more valid for observers, while they filter out other conflicting cues to maintain a consistent impression of the presenter. The latter two strategies, related to attribution theory (Weiner, 1985; Jones and Nisbett, 1987), reflect how observers look for (a) external/situational causality or (b) internal causality to explain why the presenter posted expressions that contradict his/her authentic representation in the first place.

### Origin of perceived inconsistency: Deviation from authentic representation

The first main theme involves how observers define inconsistent expressions on social media. In line with previous literature, two sets of social cues are viewed as inconsistent only when they cause cognitive dissonance for observers and drive observers to resolve such inconsistency (Slovic, 1966; Hampson, 1998). According to participants’ narratives, such cognitive dissonance usually occurs when conflicting expressions on social media threaten their observations of a presenter’s authentic aspects (such as authentic identity, personal characteristics, inner beliefs, or true feelings). That said, perceived (in)consistency on social media originates from expressions that violate or deviate from the authentic representation of a target. Being aware that self-presentation on social media can be manipulative, participants first looked for cues that could represent a presenter authentically, including who the person really was (e.g., authentic identity or true personality) or what the presenter’s true thoughts were (e.g., beliefs or true feelings). They then evaluated whether or not the presenter’s other expressions were consistent with his/her authentic representation. Moreover, the results revealed that participants adopt different selection criteria to determine a presenter’s authentic representation across contexts; thus, the following three conditions are discussed separately: (a) inconsistency within the same social account; (b) inconsistency between public and private social media accounts; and (c) inconsistency between online and offline.

In the first condition, inconsistent expressions are spotted within a single social media account. Most participants claimed that discrepancies between a presenter’s behavioral performances and beliefs among their social media posts would trigger feelings of inconsistency. On one hand, when viewing a target person’s social media homepage, participants normally relied on posts that reveal one’s beliefs or deep feelings to establish a base-rate impression, as such expressions were believed to truly represent that person. On the other hand, they expected the person’s behavioral performances to correspond with his/her beliefs because one’s behaviors should be a genuine reflection of his/her beliefs (Harter, 2002). As a result, participants experienced cognitive disequilibrium when a presenter’s behavior did not match their authentic thoughts (i.e., beliefs, general values). Reflecting on this behavior-belief inconsistency, Participant 4 in Group 2 (G2P4) (female, 21) shared an example concerning her friend:

I saw my “environmentalist” friend being inconsistent, at least I thought she should be [environmentalist], cause she posted and reposted lots of articles supporting pro-environmental behaviors on FB, like taking public transportation or never using animal products. The funny thing is, a few days ago, I saw she posted a pic [in which she] held a leather purse and said, “new bag, yay!” To me, it’s like her action is totally opposite to her value[s].

Participants judged whether an online post reflected a presenter’s true thoughts by observing the post’s length and the number of similar posts. In the G2P4 example, the participant labeled her friend an “environmentalist” as she posted and reposted lots of related articles. G3P2 (male, 21) stated, “When someone posts quite a lot or quite often about certain things, then you definitely know that is the thing he truly or deeply believes in.” That is to say, observers do not perceive all posts or cues equally, and only those that meet certain criteria (e.g., occur several times, elaborated in detail) are deemed fundamental expressions that can represent a person.

Therefore, it is worthwhile to note that our participants did not view self-contradictory posts that occurred at a superficial level as “truly” inconsistent expressions unless the inconsistency shook the fundamental expressions that were deemed to reflect a presenter’s beliefs, values, and inner feelings. In a repeatedly mentioned case, detailed by G1P1 (male, 24), a guy claimed that he intended to keep fit for an extended period of time (e.g., 1 year), yet posted pictures of himself enjoying high-fat foods only 1 or 2 days later. Our participants believed that no one could avoid such conflicting claims because it was common for people’s daily expressions to vary over time. Considering that these seemingly contradictory posts occurred at the superficial level of one’s expressions and did not reflect their inner thoughts, participants simply labeled such inconsistency “behavioral variability” or “occasional blunders.” Therefore, participants did not experience any cognitive disequilibrium or get involved in any subsequent reconciliations after viewing such posts.

The second condition of perceived inconsistency happens when a presenter’s expressions differed across their public and private accounts on the same social media platform (e.g., Instagram). For young people today, owning two social media accounts–one public and one private–is not unusual, as they can opt for different presentation settings to satisfy different needs. On public accounts, on which users provide identifiable information (e.g., real names, occupations, and affiliations with social groups) and are connected to a large number of followers, people purposefully tailor their posts to build a positive image to meet social expectations. Conversely, a private account, through which people connect with a small group of close friends and conceal their social identities by using aliases, is used to document daily life and unadulterated personal feelings (including both positive and negative moods).

Almost all participants believed that the expressions on private accounts were more authentic because one normally revealed his/her inner aspects or actual life there. Meanwhile, participants found that presentations on public accounts were comparatively more superficial and homogeneous (mostly about food, travel, or other general interests), and they closely associated such content with objectives like “showing off social ranking,” “competition,” or “wanting people to [feel] envy.” According to an observation from G2P5 (female, 22), “people always present the same thing, like nice food or travel adventure[s]. You cannot tell who the person really is. People are just a product there.” Resultantly, participants sensed inconsistency when they found conflicting expressions between these two accounts, as G6P3 (female, 21) stated:

For their main [public] account, they show they are having fun, but, in their private account, they would say, “I’m depressed, this thing is happening, blah blah.” I think it is incongruent in a sense.

In this condition, the perception of inconsistency originates from the discrepancy between the desirable identity or positive image one wants to build on a public account and the real thoughts that one expresses on a private account. For instance, G1P1 (male, 24) identified himself as a part-time gym trainer on his public Instagram account, where he claimed that “you must eat clean to keep fit” to meet people’s expectations of a qualified trainer. However, he revealed his actual life on a private account, where he stated, “I only eat salad for my breakfast, but, for the rest, I post all unclean meal[s], all rubbish food.” Apparently, participants perceived such conflicting expressions as inconsistent because the presenter’s actual behaviors (e.g., posts on the private account) violated their presumed performance/image of him as a good trainer. That said, participants viewed one’s expressions on a private account as authenticity markers to compare with the expressions on a public account, so any presentations on the public account that disagreed with these presumed authentic expressions naturally made them feel incongruent.

Under the third condition, the perception of inconsistency is caused by the discrepancy between one’s online and offline performances. Most of the time, participants were inclined to view a presenter’s offline performances as authentic because they could interact directly with and know the presenter in person offline. Therefore, when a presenter’s online expressions contradicted his/her offline persona, a sense of inconsistency naturally occurred. A typical example could be a normally introverted, “geeky” man in real life who turned out to be very narcissistic on social media and posted selfies with obvious filters. G6P5 (male, 23) described a similar case concerning his friend:

The guy is very quiet and passive offline. But online, like on Twitter, he’s very chatty, always sharing opinions about political or social issues like cyberbullying. That is kind of [an] inconsistency for me, as the online performance is not consistent with his traits in real life.

Participants usually felt confused when seeing someone they knew well (e.g., friends or familiar others) behaving differently online vs. offline, so they were inclined to find ways to justify such inconsistency. They explained that the personal traits of their friends or familiar others could be diverse and multifaceted, so this type of incongruence existed mainly because their friends used social media to showcase certain aspects of their personalities.

Online-offline inconsistency also could be observed among acquaintances. According to G1P3 (female, 20), such situations are common in online dating: “[I]t’s like he’s your ideal type on social media, but suddenly, it breaks your illusion when you come to know him [offline].” This case showed that participants barely formed a favorable impression when they saw an acquaintance behaving differently in real life vs. online because they believed that the acquaintance’s online presentation was manipulative and fabricated. These two cases reflected that people viewed offline cues mainly as authenticity markers (regardless of friends or acquaintances), but they adopted different approaches to reconciling the inconsistency that happened with friends vs. with acquaintances, which will be illustrated in detail in the following section.

### Strategies for reconciling incongruence

The second main theme concerns the strategies people adopt to reconcile inconsistent expressions on social media. As mentioned before, perceived inconsistency triggers cognitive dissonance, which pushes observers to reconcile the contradictions and eventually form a stable impression of the presenter. Three types of strategies were extracted from participants’ narratives: relying on authentic representation; perspective-taking to compensate for situational factors, and inferring inner motives behind inconsistent behaviors. By relying on more authentic information, observers filter out conflicting information and keep a coherent impression of the presenter. Through perspective-taking, observers attribute inconsistency to external causes, which would not affect the presenter’s interpersonal evaluation. However, by inferring inner motives, observers attribute inconsistency to dispositional causes and make further interpersonal evaluations of the presenter by judging the legitimacy of the causes.

The first reconciliation strategy–relying on authentic representation–refers to that observers attach greater credence or higher warranting value to cues that reflect a presenter’s authentic aspects and devalue other conflicting information. According to our participants, when they perceived that a piece of information deviated from a presenter’s authentic representation (i.e., base-rate impression), they were likely to stick to the base-rate or initially formed impression and neglect the newly captured information to maintain a coherent impression of the presenter. As noted in the previous section, the criteria for determining authentic cues vary by conditions. Generally, when reconciling perceived inconsistency within the same social media account, cues that reveal a person’s identity, personality, beliefs, and inner feelings were believed to be authentic. However, when reconciling perceived inconsistency across different contexts (public vs. private and online vs. offline), cues in private accounts or offline settings were naturally perceived as more authentic than those posted in public accounts. For example, participants claimed that they could know a more “personal human being” in private accounts because it is presumed that a presenter has no reason (as they are invisible to a large audience) to manipulate or modify their posts there. That said, participants normally attached a higher level of warranting value to information in private accounts, whereas they did not take presentations in public accounts into serious consideration.

In most cases, this strategy works better when a presenter is the observer’s familiar others (e.g., friends, family), as the observer has a greater chance of accessing the presenter’s authentic expressions in private accounts or offline in real life. G4P2 (female, 24) mentioned the case of her sister:

Like, for my sister, she’s a photographer, [and] on her main [public] account, she posts very edited photographs, cause she wants to promote herself. But on her private [account], she’s super sarcastic, and she talks with no filter with her close friends. […] It’s who she really is, [so] I enjoy her private account a lot more.

The second strategy used to reconcile incongruent impressions is perspective-taking–understanding the cause of perceived incongruence from the presenter’s perspective. Although contextual cues are absent in CMC contexts, participants attempted to compensate for missing situational factors (e.g., bad weather or other people’s invitations) by putting themselves into the presenter’s situation or referencing their own experiences, as expressed by G5P3 (female, 20): “One of my principles is putting myself into others’ shoes.” Through perspective-taking, participants attributed a presenter’s inconsistent expressions to situational elements or external causality. As long as the incongruence could be justified by external factors, participants could maintain a coherent impression of the presenter without making negative judgments.

Similar to the first strategy, participants mainly employed this strategy to address perceived inconsistency that familiar others (e.g., friends or people from their social circle) generated because the familiarity and commonalities between them made it possible to compensate for missing contextual factors. In addition, participants were more willing to show empathy and understanding toward friends and close ones. For example, when discussing friends who claimed to study hard for the final exam while still going out to party frequently, some participants admitted that they behaved similarly. G1P1 (male, 24) stated that he violated his determination to be “working hard” several times because he was invited to parties and did not want to spoil the inviters’ moods. He defended for his friends: “I think they [his friends] have reasons to do certain things at that given condition, [so] it [performing inconsistently] is really not their fault.” However, participants struggled to come up with possible situational explanations for the inconsistencies that their new connections or acquaintances from different social circles expressed. According to G4P7 (female, 20), “for people I don’t know a lot, then I don’t know why he posted inconsistently on social media. So, I just see it, and I’ll feel this person is kind of dodgy.”

The third strategy–inferring inner motives for a presenter’s perceived inconsistency–is used when observers cannot or are unwilling to think of situational explanations for people whom they do not know well when inconsistency occurs. Considering that the motives for actions are viewed as closely related to internal causality (Jones and Nisbett, 1987), inferring inner motives involves seeking dispositional causes for why people act incongruently. Through this strategy, participants rationalized the inconsistent performance of a presenter (usually an acquaintance), so they either maintained or adjusted the evaluation of the presenter’s impression depending on the motive’s legitimacy.

Due to the absence of contextual cues and personal understanding of an unfamiliar presenter, participants primarily relied on social categorical cues (i.e., information that reflects one’s social identity or belonged social group) to infer the presenter’s motives, as such cues set a normative expectation for the evaluation of people’s behaviors. This approach was evident when perceiving expressions of self-promotion. Participants mentioned that many micro-celebrities or social media influencers posted fantastic lifestyle pictures on Instagram, but lived normal lives offline. They inferred that these presenters’ motives for being online-offline inconsistent were driven by their identities as influencers. G3P2 (male, 21) stated:

I know some sports people in Singapore. They post a lot of cool or fancy photographs about themselves, like doing their sports or gathering in very, very nice places. But, in their offline life, they are quite different from what they post. They are just normal people. So, I guess they [micro-celebrities] just want to get sponsorship, and I wouldn’t expect them to always behave in the same way [online and offline] because, in a sense, their roles require them to behave inconsistently.

Participants accepted inconsistency under this circumstance because they knew that micro-celebrities engaged in such behavior “to make a living, [and] pretending to be fantastic is part of their life” (G2P5, female, 22). In other words, micro-celebrities motives for performing inconsistently did not violate the common attributes of their belonged social groups. In contrast, participants found it difficult to accept their peers’ (i.e., college students) extravagant lifestyle pictures on Instagram. When seeing posts of their peers showcasing a fancy lifestyle, participants compared such online expressions by their peer acquaintances to the expected lifestyle of a college student (the presenter’s social identity). Benchmarked against normative expectations of college students’ expenditures, they concluded that the target’s presented lifestyle did not fit the category of “college student,” and the presenter’s self-promotion was viewed as motivated by a desire for public attention.

Therefore, when processing perceived inconsistency by unfamiliar others, participants speculated the internal reasons for inconsistent expressions and could accept a certain level of inconsistency as long as the presenter’s motives were in line with his/her primary social identity. Indeed, they devalued the motive of achieving popularity or, broadly speaking, seeking external approval. When driven by such motives, a presenter’s inconsistent expressions were seen as “fake” or fabricated, leading to negative evaluations of the presenter.

## Discussion

### A two-stage process of perceiving inconsistency

**FIGURE 1:**
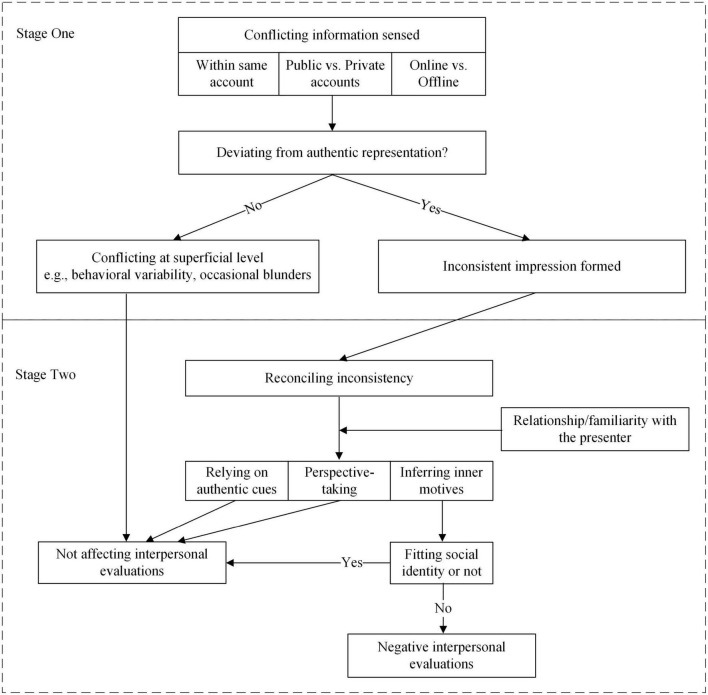
A two-stage model of processing inconsistent information in CMC.

This study aimed to (a) examine the nature of inconsistent expressions on social media from observers’ perspective, and (b) understand how observers resolve perceived inconsistency and form a coherent impression of a presenter. Two main themes were uncovered accordingly: the origin of perceived inconsistency on social media and strategies for reconciling it. In essence, the two uncovered themes reflect a two-stage process of perceiving inconsistent information on social media: The first theme describes in what circumstances observers attain a sense of inconsistency, and the second theme uncovers how observers resolve the perceived inconsistency (see Figure 1). Moreover, this study revealed that several factors–including perceived authenticity, social categorical cues, and relationship/familiarity between observers and a presenter–play a role in this two-stage process and work together in affecting observers’ interpretations of inconsistent expressions.

To explain the mixed outcomes of inconsistent information, the two-stage model of processing inconsistency uncovers a complicated mechanism of inconsistent expressions’ influence on forming interpersonal impressions. First, only when conflicting information deviates from one’s authentic representation does a sense of incongruence come into being (as in stage one). Furthermore, perceived inconsistency does not necessarily lead to negative evaluations, and the outcomes depend on how observers reconcile the inconsistency (as in stage two). To be specific, the adoption of different reconciliation strategies is subject to the relationship/familiarity between observers and a presenter. On one hand, to justify inconsistency generated by presenters like friends and familiar others, observers either mainly rely on the authentic representation and ignore conflicting information, or seek external causes through the perspective-taking strategy, both of which help them maintain a stable and favorable impression of their friends. On the other hand, when processing inconsistent expressions by acquaintances or unfamiliar others, observers turn to infer the motives for acting inconsistent and judge the legitimacy of the motive based on the presenter’s belonged British social group, and illegitimate motives (i.e., motives that are perceived to be not in line with social identity) could affect interpersonal evaluations negatively.

### Theoretical implications for online interpersonal perceptions

By investigating inconsistent expressions on social media, this study suggests that the interdependence–not only complementary but also a conflicting relationship–between social cues conveys valuable meaning. In short, inconsistency *per se* is informative in computer-mediated interpersonal perception. As shown in the proposed two-stage model, perceived inconsistency triggers distinct perception paths of integrating available cues and results in different outcomes in social evaluation. With this said, the present study–which connects attribution theory with warranting theory and the authenticity model in CMC–enhances our understanding of computer-mediated interpersonal perception.

This study highlights the central role of perceived authenticity in online interpersonal perception. Considering the surge of misinformation in current online contexts, increasing research attention has been given to the role of authenticity (Marwick and Boyd, 2011; Wotipka and High, 2016; Lee, 2020; Tang et al., 2020). Recent research has proposed that the perception of authenticity is bonded with the sense of (in)consistency concerning observers’ base-rate knowledge (Lee, 2020), while the present study further suggests that an interrelationship exists between (in)consistency and authenticity. That is authentic representation functions as a gauge through which observers can evaluate whether or not a presenter’s expressions are consistent and make interpersonal evaluations. The results indicate that whether a presenter’s (mostly acquaintances) inconsistent expressions jeopardize their interpersonal evaluations essentially depends on whether the feeling of authenticity is threatened (i.e., the third reconciliation strategy). Furthermore, this study also unveiled how observers sought authenticity markers on social media. As shown in the first theme, social cues that are believed to reflect authentic aspects of a presenter include (a) those on private accounts or generated in offline settings, as opposed to public accounts, and (b) those revealing one’s identity, personality, beliefs, and inner feelings, even when they are on public accounts. These findings collectively confirmed perceived authenticity’s significant impact in CMC, which deserves future research attention.

Meanwhile, a theoretical connection between the concept of authenticity and warranting theory is built in this study. The results showed that observers usually count on perceived authenticity to assess self-generated cues’ validity and warranting value. As illustrated in the first reconciliation strategy, observers attach greater credence or higher warranting value to cues viewed as reflecting a presenter’s authentic self, while they simultaneously devalue cues that are deemed fabricated. That said, authentic cues may possess relatively higher warranting value because such cues are perceived to be less prone to manipulation. Recent research regarding warranting theory has started to shift its emphasis toward assessing the warranting value of cues from the same information source (e.g., a source’s obfuscation level; DeAndrea and Carpenter, 2018). The findings of the present study thereby add to this body of knowledge by proposing that perceived authenticity of an information source can be viewed as an indicator of warranting value.

This study also extends the application of attribution theory to demonstrate the perception of inconsistent information in CMC. Parallel to explaining inconsistency in offline contexts, the present study revealed that people also adopt the asymmetrical manner to make attributions for online inconsistent expressions generated by friends or familiar others vs. those generated by acquaintances. As discussed, despite the limited contextual cues online, people can compensate for the missing situational factors via the perspective-taking strategy. In this way, they can avoid judging their friends’ personalities or personal traits to maintain a favorable impression of and a satisfactory relationship with friends (see Prentice, 1990; Malle, 2006). When inconsistency occurring with acquaintances, however, people make dispositional attributions directly (i.e., judge personalities) by inferring their inner motives. The results confirmed that people de-individualize acquaintances and oversimplify the motives for their actions in CMC (Reicher et al., 1995), as they rely on social categorical cues (i.e., information reflecting one’s social identity or social group) to speculate an acquaintance’s motives and judge such motives’ legitimacy. These findings suggest that the relationship or familiarity between a presenter and observer saliently affects how observers attribute non-normative and unexpected behaviors in online contexts.

## Conclusion

Before concluding, a few limitations in the present study should be noted to provide inspiring suggestions for future research. Regarding the participants, our findings were mainly drawn from the narratives of people in Singapore, which limit the scope of our interpretation of perceived inconsistency on social media. Participants in this study are dominantly subject to the specific socio-cultural values in Asian societies. Influenced by the collectivist culture, they are likely to adhere to social norms when using social media (Na et al., 2015), so they may be more sensitive to observing and reconciling non-normative information such as inconsistent expressions. Future research should entail similar interviews in countries with different cultural backgrounds, as demographics and culture may play a role in the recognition and reconciliation of online inconsistency. For instance, future studies can explore how individualist vs. collectivist socio-cultural values affect social media users’ perception, reaction, and explanation to the perceived (in)consistency, and introduce socio-cultural factors into our proposed two-stage model. Regarding certain methodological concerns, we examined how people generally perceive inconsistent expressions but did not prime our participants with specific scenarios (e.g., using experimental stimulus) to make a discussion. Thus, although we discovered certain factors involved in the process of perceiving inconsistency, we may have missed other important factors (such as personal relevance or the motivation of accuracy) that take effects in this process, which deserve further examination. Indeed, we focused on the perceptions of inconsistency in non-interactive online settings (e.g., profile viewing) in this exploratory study; however, people also directly and continuously interact with others on social media. Recent research posits that the negative effects of online presentation inconsistencies may diminish in interactive settings (like a short conversation of 30 min) (Tang et al., 2020). Future research thus can uncover the mechanism of long-term effects of perceived inconsistency in continuous interactions. Regarding the implications, the present study attempted to make contributions to the field of online interpersonal interactions by uncovering the significance of perceived (in)consistency and authenticity in personal impression formation and impression management. Notably, consistency and authenticity are also considered significant factors when building appropriate and attractive corporation images on social media (Marwick and Boyd, 2011). Social media has become a common venue for corporations to improve brand images, and manage customer relationships and public relationships globally (Gavurova et al., 2018; Matkevičienė and Jakučionienė, 2021). To extend the findings of this study, future research should examine the influence of perceived (in)consistency for corporations’ social media accounts and how it affects the customers’ impressions concerning different corporations.

Through examining inconsistent expressions on social media, the present study develops a novel model of processing perceived inconsistency, which makes theoretical contributions to the literature on online interpersonal perceptions. First, by proposing that the sense of authenticity functions as a crucial gauge in perceiving inconsistency, this study highlights authenticity’s significant role in CMC (Lee, 2020). Second, by suggesting that authenticity can help observers evaluate warranting value among self-generated cues, this study connects the notion of authenticity with warranting theory, thereby advancing the notion of this theory (Walther and Parks, 2002). Finally, the process of computer-mediated interpersonal perception is not completely objective, because observers subjectively interpret the meaning of the same set of social cues and make asymmetrical attribution depending on their relationship or familiarity with the presenter. These contributions are meaningful in the current era of social media when interpersonal rapport is heavily dependent on impressions formed via CMC.

## Data availability statement

The original contributions presented in this study are included in the article/supplementary material, further inquiries can be directed to the corresponding author.

## Ethics statement

The studies involving human participants were reviewed and approved by National University of Singapore. The patients/participants provided their written informed consent to participate in this study.

## Author contributions

PL designed, conducted, and wrote the manuscript. HC and YQ co-designed and collaborated on wrote the manuscript. All authors contributed to the article and approved the submitted version.

## Appendix | Focus group guide

- Please recall your experiences of viewing others’ profiles on social media, like, on Facebook or Instagram or any other social media platforms, and please describe the situation(s) that leads you to have a feeling of inconsistency about the presenter (including your friends/family members or acquaintances, social influencers, opinion leaders or even yourself).
  ◦ Guiding example: for instance, you may see your friends, or acquaintances, or even social influencers sometimes post on their timeline inconsistently, in other words, saying something 1 day, while opposing to it the other day.
  ◦ Why do you consider the example you just mentioned as inconsistent? Put differently, how would you define a set of expressions as inconsistent?

- How do you usually notice the inconsistent expressions? or under what conditions, the inconsistent expressions are noticeable for you?
- How do you react to these incongruent expressions? For instance, will you reconcile or try to resolve the perceived inconsistent information?
  ◦ If “yes,” ask the following questions:
    How or through which way would you reconcile/address the inconsistency?
  What factors may drive you to reconcile/rethink about the inconsistency? (or ask participants to describe how you reconciled the inconsistent expressions generated by your friends/family members or by acquaintances, social influencers, or opinion leaders).
  ◦ If “no,” ask why you do not make any reconciliation?

- Will the perceived inconsistency affect your overall evaluation about the presenter or further interaction with the presenter?
  ◦ If “yes,” how or in what way does it affect the evaluation?
  ◦ If “no,” why it does not affect?

- Think about yourself, have you ever disclosed inconsistently? Did anyone point out your inconsistency? How did you deal with that situation?
- Of all the things we’ve discussed today, what else would you say about inconsistent expressions on social media?
